# Three-Year Outcomes After Surgical Aortic Valve Replacement With a Bioprosthetic Valve from the Multi-Centre IMPACT Registry

**DOI:** 10.1093/icvts/ivag056

**Published:** 2026-03-05

**Authors:** Farhad Bakhtiary, Peter Benedikt, Nikolaos Bonaros, Michael Borger, Mirko Doss, Richard Feyrer, Jürg Grünenfelder, Tamer Owais, Seymur Karimli, Ka Yan Lam, Andreas Liebold, Andreas Martens, Parwis Massoudy, Islam Batashev, Jochen Pöling, Maximilian Philipp Scherner, Justus Strauch, Matthias Thielmann, Andreas Vötsch, Thomas Walther, Alberto Weber, Stefan Wiesinger, Matthias Eden, Jürgen Kammler, Beate Botta, Nataliya Trushina, Anjaly Vijayan, Peter Bramlage, Andreas Zierer

**Affiliations:** Department of Cardiac Surgery, University Hospital Bonn, Bonn, 53127, Germany; Department of Cardiovascular and Thoracic Surgery, Medical Faculty, Johannes Kepler University Linz, Linz, 4020, Austria; Clinical Research Institute for Cardiovascular and Metabolic Diseases, Medical Faculty, Johannes Kepler University Linz, Linz, 4020, Austria; Department of Cardiac Surgery, Medical University of Innsbruck, Innsbruck, 6020, Austria; Department of Cardiac Surgery, Leipzig Heart Center, Leipzig, 04289, Germany; Department of Cardiothoracic Surgery, Helios Clinic Siegburg, Siegburg, 53721, Germany; Department of Cardiac Surgery, Clinic for Cardiovascular Surgery, Central Military Hospital, Koblenz, 56072, Germany; Department of Cardiac Surgery, Heart Clinic Zurich, Hirslanden Klinik, Zurich, 8032, Switzerland; Department of Cardiothoracic Surgery, Helios Clinic Wuppertal, Wuppertal, 42117, Germany; Department of Thoracic and Cardiovascular Surgery, University Hospital Würzburg, Würzburg, 97080, Germany; Department of Cardiothoracic Surgery, Catharina Hospital Eindhoven, Eindhoven, 5623, Netherlands; Department of Cardiothoracic and Vascular Surgery, University of Ulm Medical Center, Ulm, 89081, Germany; Department of Cardiac Surgery, Oldenburg Clinic, University of Oldenburg, Oldenburg, 26133, Germany; Department of Cardiac Surgery, Klinikum Passau, Passau, 94032, Germany; Department of Cardiac Surgery, Klinikum Nürnberg-Paracelsus Medical University, Nuremberg, 90419, Germany; Department of Cardiac Surgery, Schüchtermann-Clinic Bad Rothenfelde, Bad Rothenfelde, 49214, Germany; Department of Cardiac Surgery, University Hospital Duesseldorf, Duesseldorf, 40225, Germany; Department of Cardiothoracic Surgery, Berufsgenossenschaftliches Universitätsklinikum Bergmannsheil, Bochum, 44789, Germany; Department of Thoracic and Cardiovascular Surgery, University Duisburg-Essen, Essen, 45147, Germany; Department of Cardiovascular and Endovascular Surgery, Paracelsus Medical University, Salzburg, 5020, Austria; Department of Thoracic Surgery, Frankfurt University Hospital, Frankfurt, 60590, Germany; Department of Cardiovascular Surgery, Heart Center Hirslanden, Zurich, 8032, Switzerland; Department of Cardiovascular and Thoracic Surgery, Hospital Wels-Grieskirchen GmbH, Wels, 4600, Austria; Department of Medicine III: Cardiology, Angiology, and Pneumology, Heidelberg University, Heidelberg, 69120, Germany; Kepler University Hospital Linz, Department of Cardiology and Medical Intensive Care, Medical Faculty, Johannes Kepler University, Linz, 4020, Austria; Department of Internal Medicine II, Paracelsus Medical University Salzburg, Salzburg, 5020, Austria; Institute for Pharmacology and Preventive Medicine, Cloppenburg, 49661, Germany; Institute for Pharmacology and Preventive Medicine, Cloppenburg, 49661, Germany; Institute for Pharmacology and Preventive Medicine, Cloppenburg, 49661, Germany; Institute for Pharmacology and Preventive Medicine, Cloppenburg, 49661, Germany; Department of Cardiovascular and Thoracic Surgery, Medical Faculty, Johannes Kepler University Linz, Linz, 4020, Austria; Clinical Research Institute for Cardiovascular and Metabolic Diseases, Medical Faculty, Johannes Kepler University Linz, Linz, 4020, Austria; Department of Cardiovascular and Thoracic Surgery, Hospital Wels-Grieskirchen GmbH, Wels, 4600, Austria

**Keywords:** aortic stenosis, surgical aortic valve replacement, valve durability, INSPIRIS RESILIA

## Abstract

**Objectives:**

To investigate 3-year outcomes of surgical aortic valve replacement using the INSPIRIS RESILIA aortic valve bioprosthesis in patients with severe aortic stenosis.

**Methods:**

IMPACT registry is a prospective, multicentre, international registry with a 5-year follow-up. After 3 years, haemodynamic performance, all-cause mortality, and valve-related mortality were determined.

**Results:**

A total of 556 patients who underwent surgical aortic valve replacement with the INSPIRIS RESILIA aortic valve were enrolled between December 2019 and June 2021. The mean age was 63.4 ± 8.5 years, 29.0% were female, with a EuroSCORE II of 2.2 ± 2.5% and an STS score of 1.7 ± 2.2%. Hypertension (66.2%), coronary artery disease (34.7%), and diabetes (18.4%) were the most common comorbidities. A total of 49.3% of patients underwent full sternotomy, 58.3% isolated aortic valve replacement. The most commonly implanted valve sizes were 23 mm (35.4%) and 25 mm (30.9%). At 3 years, overall survival was 91.0%, freedom from valve-related mortality was 97.5%, from prosthetic endocarditis 96.0%, from stroke 91.5%, from valve-related dysfunction 98.0%, from reintervention 96.2%, from structural valve deterioration stage 2 96.8%, and from stage 3 99.4%. The mean transvalvular gradient was 11.9 mmHg at 3 years, with an indexed effective orifice area of 0.8 cm^2^/m^2^. Functional class improved from 41.2% class III or IV at baseline to 5.7% at 3 years.

**Conclusions:**

Three-year outcomes, including survival and haemodynamic and functional status after surgical aortic valve replacement using the INSPIRIS RESILIA bioprosthetic aortic valve, were reported in a real-world patient population with severe aortic stenosis.

**ClinicalTrials.gov:**

NCT04053088.

## Graphical abstract

**Figure:**
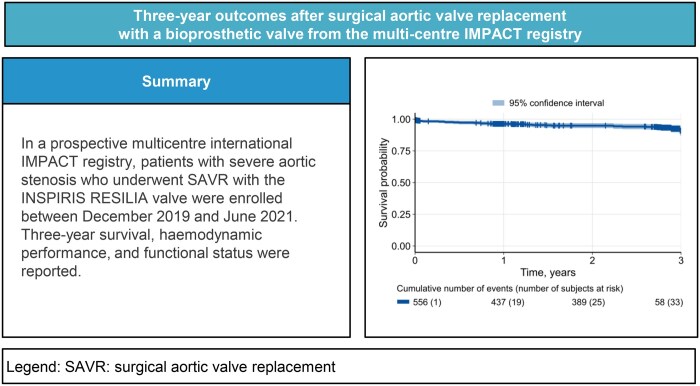

## INTRODUCTION

Aortic valve (AV) intervention remains a standard choice for severe aortic stenosis (AS), with surgery recommended for patients under the age of 70.^1^ As for the surgical valve type, the 2020 American guidelines recommend mechanical valves for patients <50, mechanical or biological for ages 50-65, and bioprosthetic valves for >65,^2^ while the 2025 ESC/EACTS guidelines recommend a mechanical valve in patients younger than 60 years and a bioprosthesis above 70 years, meaning that surgical valve type is up for discussion for patients of quite a wide age range.^1^ As modern bioprosthetic valves demonstrate good performance and durability, reasonable reoperation rates, and avoid lifestyle implications of long-term anticoagulation,^3^^,^^4^ patients may opt for a biological prosthesis, but the risk of structural valve deterioration (SVD) persists.^5^ To reduce the incidence of SVD, the INSPIRIS RESILIA (Edwards Lifesciences) aortic valve (AV) was developed. This stented bioprosthetic, tri-leaflet valve, comprised of bovine pericardial tissue, utilizes integrity preservation technology to enhance the tissue’s anti-calcification properties, potentially reducing SVD incidence.^6^^,^^7^

Several studies on the INSPIRIS RESILIA bioprosthesis have shown good early survival rates and haemodynamic performance in patients with severe AS.^8–10^ The COMMENCE trial demonstrated excellent safety of a biological valve with RESILIA tissue, with no SVD cases reported up to 5 years,^11–13^ and 99.3% freedom from SVD at 7 years.^14^ The INSPIRIS RESILIA Durability (INDURE) registry also indicated good safety and improved quality of life, with no SVD among patients younger than 60 years.^15^

Patients with severe AS who require surgical intervention often have various pre-existing comorbidities, highlighting the need for evidence on the clinical performance of the newest bioprostheses, like the INSPIRIS RESILIA valve, in diverse populations. Thus, the IMPACT registry aims to provide complementary data on the safety and efficacy of surgical aortic valve replacement (SAVR) with this bioprosthesis in real-world patients with heterogeneous clinical backgrounds. The primary end-point is all-cause mortality, with secondary end-points including cardiac-related and valve-related mortality, haemodynamic performance, durability, and other outcomes in patients with chronic kidney disease, diabetes, hypertension, metabolic syndrome, or chronic inflammation. This article reports on the 3-year outcomes of the IMPACT registry.

## METHODS

IMPACT (ClinicalTrials.gov: NCT04053088, https://clinicaltrials.gov/study/NCT04053088, first submitted 06.08.2019) is a non-interventional, prospective, open-label, multicentre, international registry of patients undergoing SAVR with an INSPIRIS RESILIA (Edwards Lifesciences)^16^ valve across 21 sites in Germany, Austria, Switzerland, and the Netherlands between December 2019 and June 2021. Ethics committees at each site granted approval (approval for lead sites were granted on November 14, 2019 [Siegburg, #2019350] and December 9, 2019 [Linz/Wels, #1190/2019]), and patients provided written informed consent before enrollment. The registry is conducted in accordance with the European Medical Device Regulation (2017/745 of April 5, 2017), ISO 14155:2011 and the Declaration of Helsinki. The registry was supported by Edwards Lifesciences (Nyon, Switzerland) with a research grant to the sponsor IPPMed GmbH (Cloppenburg, Germany).

### Patients

Consecutive patients ≥18 years undergoing SAVR receiving the INSPIRIS RESILIA valve were included with the following criteria: planned SAVR with/without concomitant ascending aortic root replacement and/or coronary artery bypass graft based on a pre-interventional evaluation and device instruction, excluding off-label techniques. Patients had yearly on-site follow-ups. Exclusion criteria were: (1) active endocarditis or myocarditis (or ≤3 months prior); (2) valve implantation not possible per device instructions (eg, damaged valve, compromised the packaging integrity, the device has expired, or correct sizing cannot be achieved); (3) life expectancy <12 months; and (4) pregnancy at surgery.

### Objectives

The primary objective was to assess 3-year all-cause mortality post-SAVR. Secondary objectives included evaluating cardiac/valve-related mortality, haemodynamic valve deterioration (HVD), haemodynamic performance, durability, New York Heart Association (NYHA) class changes, and clinical outcomes. Valve-related safety outcomes were assessed using standardized Valve Academic Research Consortium (VARC)-2 criteria (repeat procedure for cardiovascular reasons, prosthetic valve endocarditis, thrombosis, and thromboembolic events)^17^ along with additional predefined outcomes, including valve-related dysfunction, permanent pacemaker implantation (PPI), and HVD. HVD was defined by VARC-3 criteria^18^ as moderate (stage 2) or severe (stage 3), based on increases in transvalvular gradients with concomitant reductions in effective orifice area and/or Doppler velocity index, or the worsening of intraprosthetic aortic regurgitation.

### Statistical analysis

All patients underwent successful implantation of the INSPIRIS RESILIA valve. Analyses were conducted on an as-treated basis. Data were analysed using descriptive statistics, with estimands defined as proportions for categorical variables, measures of central tendency for continuous variables, and time-to-event probabilities for survival and valve-related events. Categorical variables presented as counts (*n*) and frequencies (%), and continuous variables as mean (standard deviation) or median (interquartile range). Normality was assessed with the Shapiro-Wilk test. Group differences were assessed using linear models for continuous outcomes and generalized linear models for categorical outcomes. Paired comparisons versus baseline used Wilcoxon signed-rank and McNemar’s tests, respectively. Kaplan-Meier methods were used to estimate survival probabilities and event-free outcomes, with freedom from events (FFEs) defined as the probability of remaining free from the event of interest over time. Comparisons between age groups (<60 vs ≥60 years) were performed using the log-rank test. Valve-years were calculated as the cumulative follow-up time, defined as the sum of individual patient follow-up durations from valve implantation to the last available follow-up or three-year cutoff. Patients who died or were lost to follow-up were included in time-to-event analyses and censored at the time of death or last available follow-up. Effect estimates are reported with 95% confidence intervals. Time to repeat procedure was additionally analysed using cumulative incidence functions accounting for competing risks. No imputation was performed; missing values were excluded from analyses for the respective variables. A *P*-value of <.05 was considered significant. Analyses were performed using R (https://www.R-project.org/).

## RESULTS

**Figure 1.**
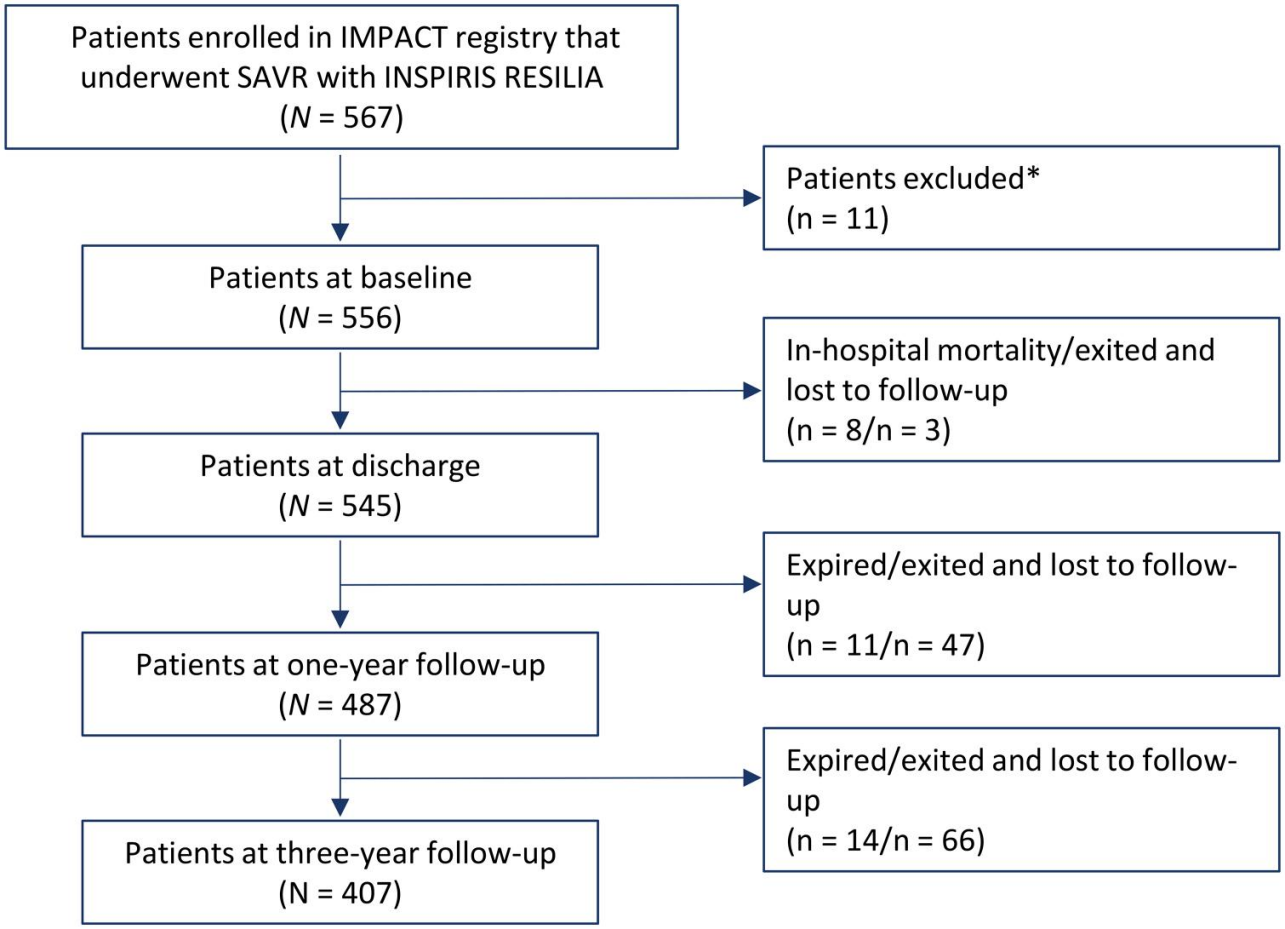
Patient Flow Chart. *n = 6 patients received another valve type, *n* = 2 had endocarditis before intervention, *n* = 1 had preoperative stroke, *n* = 1 refused to return for follow-ups, *n* = 1 withdrew consent

In the IMPACT registry, 567 patients underwent SAVR with the Edwards INSPIRIS RESILIA AV; 556 patients met the inclusion criteria for the present analysis (**Figure 1**). The mean follow-up duration was 2.6 ± 0.8 years.

### Patient characteristics

Patients (29% female) had a mean age of 63.4 years and a BMI of 28.4 kg/m^2^ (**Table 1**), with 32.2% patients having BMI > 30. The mean EuroSCORE II of the patients was 2.2%, and the STS risk score was 1.7. Notably, 229 patients (41.2%) had NYHA class III/IV, and 31 (5.6%) had Canadian Cardiovascular Society (CCS) grade III/IV angina. Common comorbidities included hypertension (66.2%), coronary artery disease (34.7%), and type II diabetes mellitus (16.2%). Baseline echocardiography showed that 397 patients (71.4%) had severe AS and 67 patients (12.1%) had severe AV regurgitation.

**Table 1.** Patient Characteristics

Mean ± standard deviation; or *n* (%)	*N*	Total population
Age, years	556	63.4 ± 8.5
Female gender	556	161 (29.0)
BMI, kg/m²	556	28.4 ± 5.3
NYHA class III/IV	556	229 (41.2)
CCS angina grade III/IV	556	31 (5.6)
EuroSCORE II, %	555	2.2 ± 2.5
STS risk score, %	553	1.7 ± 2.2
Medical history		
Coronary artery disease	553	192 (34.7)
Systemic hypertension	556	368 (66.2)
Prior myocardial infarction	556	26 (4.7)
Prior pacemaker implantation	556	6 (1.1)
Prior percutaneous coronary intervention	556	73 (13.1)
Diabetes mellitus	556	
Type I		12 (2.2)
Type II		90 (16.2)
Peripheral vascular disease	556	36 (6.5)
Prior stroke/transient ischaemic attack	556	24 (4.3)
Chronic obstructive pulmonary disorder	556	45 (8.1)
Creatinine, mg/dL	526	1.0 ± 0.6
Renal failure (estimated glomerular filtration rate <15 and/or dialysis)	556	12 (2.2)
Echocardiography		
Severe AV stenosis	556	397 (71.4)
Severe AV regurgitation	552	67 (12.1)
Severe pulmonary hypertension, >55, mmHg	214	12 (5.6)

Abbreviations: AV = aortic valve; BMI = body mass index; CCS = Canadian Cardiovascular Society; EuroSCORE = European System for Cardiac Operative Risk Estimation; NYHA = New York Heart Association; STS = Society of Thoracic Surgeon score.

### Procedural and hospital stay characteristics

Forty-four percent of patients had bicuspid valve morphology. Regardless of leaflet number, the majority (53.2%) presented with degenerative valve aetiology (**Table 2**). Full sternotomy was performed in 274 patients (49.3%), with upper haemisternotomy as the second most common approach (34.4%). Isolated aortic valve replacement was performed in 324 patients (58.3%). The mean valve size was 24.0 mm, with the most common size being a 23 mm valve (35.4%), followed by 25 mm (30.9%) (**Figure S1**).

**Table 2.** Interventional Characteristics and Hospital Length of Stay

Mean ± standard deviation; median (interquartile range); or *n* (%)	*N*	Total population
Valve morphology	555	
Bicuspid		244 (44.0)
Tricuspid		311 (56.0)
Valve aetiology	555	
Congenital		245 (44.2)
Degenerative		295 (53.2)
Rheumatic		1 (0.2)
Other or none		13 (2.3)
Surgical approach	556	
Full sternotomy		274 (49.3)
Right anterior mini-thoracotomy		91 (16.4)
Upper haemisternotomy		191 (34.4)
Isolated AVR	556	324 (58.3)
Concomitant procedure	556	232 (41.7)
AVR + CABG		75 (32.3)
AVR + CABG + other^a^		18 (7.8)
AVR + other		139 (59.9)
Coronary artery bypass graft	556	93 (16.7)
Number of grafts	93	
1		31 (33.3)
2		27 (29.0)
3		23 (24.7)
4		7 (7.5)
5		5 (5.4)
Number of anastomoses	556	0.4 ± 1.0
Root replacement	556	39 (7.0)
Supracoronary tube graft	556	56 (10.1)
Procedural time and implantation details		
Cross-clamp time, min	555	75.1 ± 28.9; 70.0 (55.0, 93.0)
Cardiopulmonary bypass time, min	555	110.8 ± 43.8; 103.0 (81.0, 133.0)
Total operation time (skin-to-skin), min	555	198.9 ± 72.3; 191.0 (153.0, 232.0)
Valve size, mm	556	
19		4 (0.7)
21		88 (15.8)
23		197 (35.4)
25		172 (30.9)
27		74 (13.3)
29		21 (3.8)
Implantation success	556	556 (100.0)
Visual paravalvular leak	556	3 (0.5)
Complications	556	0 (0)
Intraprocedural mortality	556	0 (0)
Hospital length of stay		
Discharged to	554	
Home		347 (62.6)
Rehabilitation unit		132 (23.8)
Other hospital		67 (12.1)
Death		7 (1.3)
Other		1 (0.2)
Hospital stay, days	554	10.3 ± 6.5; 8.0 (7.0, 12.0)
Intensive care unit stay, h	548	50.1 ± 65.3; 24.0 (21.0, 49.0)
Mechanical ventilation, h	519	16.5 ± 57.5; 7.0 (5.0, 11.0)
Patient-prosthesis mismatch degree	349	
Severe		14 (4.0)
Moderate		76 (21.8)
No		259 (74.2)

a “other” includes aortic root replacement, supracoronary ascending aorta replacement, and other concomitant cardiac procedures.

Abbreviations: AVR = aortic valve replacement; CABG = coronary artery bypass graft.

Valve implantation was successful in all patients, with no intraprocedural complications or intraprocedural mortality. A paravalvular leak was assessed qualitatively/visually at the time of the procedure after the first implantation attempt; severity grading was not collected at this time point, and all first implantations were considered successful. At discharge, paravalvular leak was mild in 6 patients (1.2%) and moderate in 2 patients (0.4%), with no cases of severe leak (**Figure S2**). No moderate or severe paravalvular leak was observed at 1- or 3-year follow-up. The mean hospital stay was 10.3 days (median 8.0), with an intensive care unit (ICU) stay of 50.1 h and a mechanical ventilation duration of 16.5 h (**Table 2**). Most patients (62.6%) were discharged home post-surgery. With BMI-adjusted thresholds as per VARC-3 criteria,^18^ moderate PPM was observed in 21.8% of patients and severe in 4.0%.

### Haemodynamic parameters, left ventricular remodelling, and anticoagulation/antiplatelet therapy

The mean AV pressure gradient decreased from 42.7 mmHg at baseline to 11.2 mmHg post-surgery, and remained stable after 3 years (11.9 mmHg, *P* < .001, **Table 3**, **Figure S3**). The indexed effective orifice area increased from 0.5 cm^2^/m^2^ at baseline and remained improved at 3 years (0.8 cm^2/^m^2^). Left ventricular ejection fraction improved from 57.6% at baseline to 59.4% at 3 years. Compared to baseline (128.5 g/m^2^), the left ventricular mass index was decreased at 3 years (102.7 g/m^2^). All aforementioned improvements were statistically significant (*P* < .001; **Figure S4**).

**Table 3.** Three-Year Changes in Haemodynamics, Left Ventricular Regression, and Anticoagulation or Antiplatelet Therapy

**Mean** **±** **standard deviation, or *n* (%)**	Baseline	Discharge	**1** **year**	**3** **years**
Mean AV gradient, mmHg	42.7 ± 18.3	11.2 ± 4.9	11.3 ± 5.1	11.9 ± 5.6
Peak AV gradient, mmHg	69.6 ± 27.9	20.4 ± 8.1	20.7 ± 9.0	22.2 ± 10.0
Peak velocity, m/s	4.1 ± 2.5	2.2 ± 1.0	2.4 ± 2.0	2.8 ± 3.0
Doppler velocity index	0.27 ± 0.16	0.54 ± 0.14	0.50 ± 0.15	0.48 ± 0.14
Effective orifice area (indexed), cm²/m²	0.52 ± 0.41	0.98 ± 0.27	0.88 ± 0.25	0.84 ± 0.25
Left ventricular ejection fraction, %	57.6 ± 10.1	56.0 ± 9.8	59.2 ± 7.9	59.2 ± 7.9
Left ventricular end-diastolic diameter, mm	49.5 ± 8.9	47.9 ± 7.2	46.5 ± 7.6	48.1 ± 7.0
Left ventricular end-diastolic volume index, ml/m^2^	60.1 ± 24.0	54.9 ± 17.9	51.7 ± 17.5	55.4 ± 19.9
Left ventricular mass index, g/m^2^	128.5 ± 40.7	122.2 ± 34.2	107.1 ± 45.5	102.7 ± 33.0
Anticoagulation	75 (13.5)	220 (39.7)	112 (24.0)	104 (25.6)
Vitamin K antagonists	15 (2.7)	89 (16.1)	14 (3.0)	12 (3.0)
New oral anticoagulants	50 (9.0)	67 (12.1)	86 (18.4)	88 (21.7)
Low molecular weight heparin	9 (1.6)	76 (13.7)	4 (0.9)	1 (0.2)
Unfractionated heparin	6 (1.1)	10 (1.8)	1 (0.2)	0 (0.0)
Antiplatelet drugs	242 (43.5)	397 (71.7)	291 (62.3)	246 (60.6)
Acetylsalicylic acid	232 (41.7)	382 (69.0)	276 (59.1)	233 (57.4)
Platelet inhibition other than acetylsalicylic acid	27 (4.9)	59 (10.6)	17 (3.6)	17 (4.2)

Abbreviation: AV = aortic valve.

Post-surgical anticoagulation use increased from baseline (13.5%) to 39.7% post-surgery and 25.6% at 3 years (**Table 3**). Vitamin K oral antagonists were commonly used post-surgery (16.1%), while their usage declined, and new oral anticoagulants (NOACs) became more common at 3 years (21.7%). Antiplatelet drug usage increased post-surgery (71.7%) and 3 years (60.6%) compared to baseline (43.5%), with acetylsalicylic acid being the most common.

### Functional status and clinical outcomes

**Figure 2.**
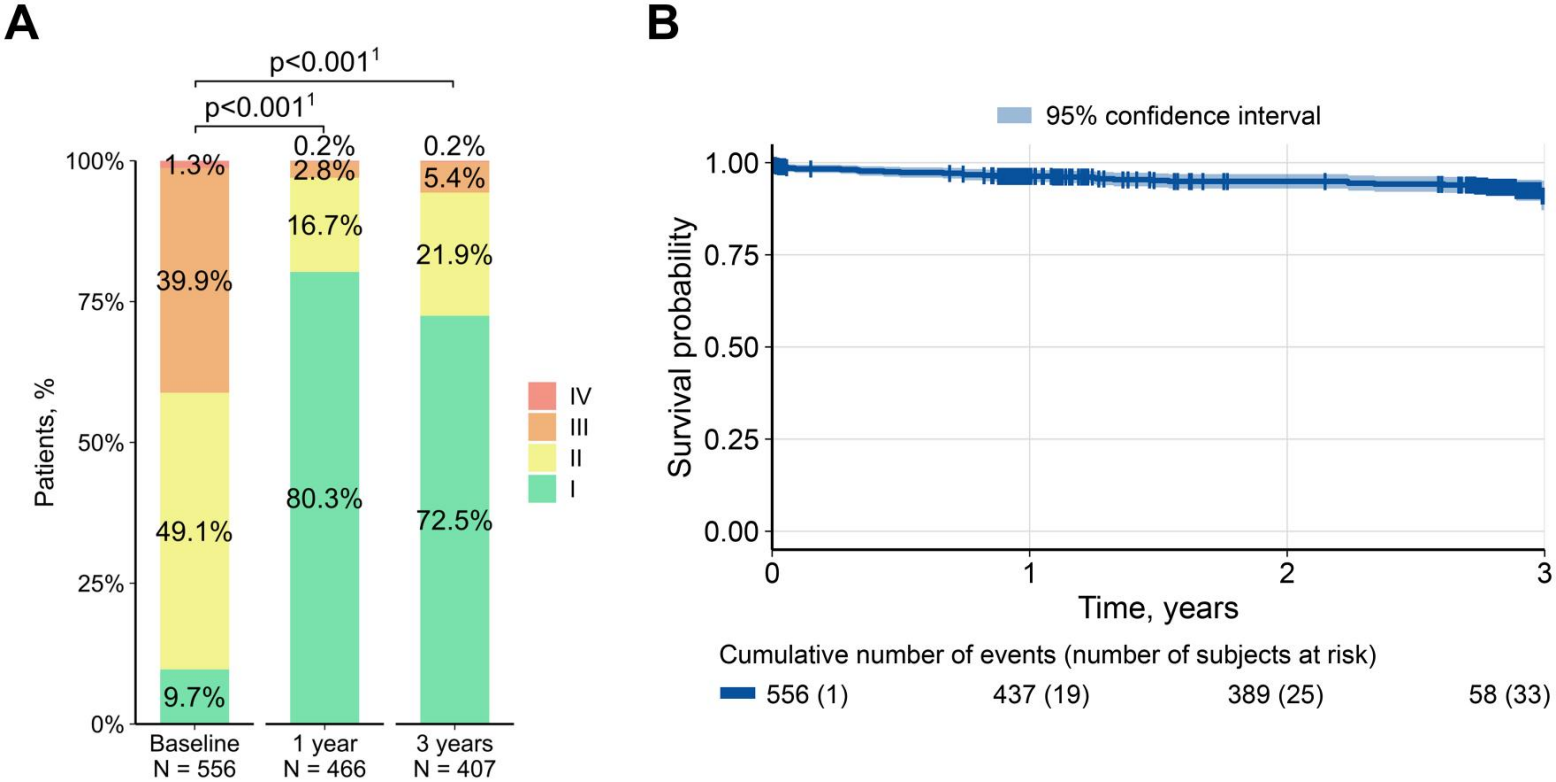
Three-year outcomes. (A) New York Heart Association class changes. (B) Survival. ^1^Pairwise comparisons for classes III/IV with random effects

There was a significant reduction in NYHA class III/IV patients at 3 years (5.7%; *P* < .001) compared to baseline (41.2%) (**Figure 2A**).

Within 30 days after SAVR, 8 patients died (1.4%) (**Table 4**). At 3 years (late outcomes >30 days; 1314.1 valve years [vy]), 25 patients (1.9%/vy) died, including 13 patients (1.1%/vy) dying of cardiovascular reasons, with 8 patients (0.7%/vy) due to valve-related reasons (**Table 4**, **Figure 2B**). Three-year freedom from all-cause mortality was 91.0% and from valve-related mortality was 97.6%.

**Table 4.** Three-Year Clinical Outcomes

	Total number of events	**Early events (≤30** **days), *n* (% of 556)**	**Late events (up to 3** **years), *n* (linearized rate/1314 valve years)**	**Freedom from events at 3** **years, % (95% confidence interval)**
All-cause mortality	33	8 (1.4)	25 (1.9)	91.0 (87.1, 95.1)
Cardiovascular-related	21	8 (1.4)	13 (1.0)	94.4 (91.1, 97.9)
Valve-related	12	4 (0.7)	8 (0.6)	97.6 (96.2, 98.9)
Endocarditis	14	1 (0.2)	13 (1.0)	96.4 (94.2, 98.6)
Valve thrombosis	0	0 (0.0)	0 (0.0)	100.0 (100.0, 100.0)
Stroke/transient ischaemic attack	24	19 (3.4)	5 (0.4)	95.5 (93.7, 97.3)
Stroke	17	16 (2.9)	1 (0.1)	96.9 (95.4, 98.3)
Valve-related dysfunction	7	2 (0.4)	5 (0.4)	98.2 (96.9, 99.6)
Requirement for repeat procedure	11	3 (0.5)	8 (0.6)	96.7 (94.5, 99.0)
Permanent pacemaker implant	34	24 (4.3)	10 (0.8)	93.5 (91.3, 95.7)
HVD stage 2	6	0 (0)	6 (0.5)	97.7 (95.8, 99.7)
HVD stage 3^a^	1	0 (0)	1 (0.1)	99.5 (98.6, 100.0)

a One patient had a valve-in-valve procedure; valve size is not available.

Abbreviation: HVD = haemodynamic valve deterioration.

From 30 days to 3 years, 13 patients (1.0%/vy) had prosthetic valve endocarditis, 5 patients (0.5%/vy) had thromboembolic events, and 1 patient (0.1%/vy) had a stroke. Valve-related dysfunction was reported in 5 patients (0.4%/vy) and 8 patients (0.6%/vy) required repeated procedures. Ten patients (0.8%/vy) underwent PPI and 6 patients (0.5%/vy) had moderate (stage 2) HVD. One patient (0.1%/vy) had developed severe HVD (stage 3) at the 3-year follow-up, associated with echocardiographic evidence of leaflet thickening and reduced leaflet mobility.

No age-related (<60 vs ≥60 years) differences were observed for most outcomes (**Table S1**). However, patients <60 years showed a higher cumulative incidence of repeat procedure (defined as valve-related reintervention performed after the index surgery) (Gray’s test *P* = .017; **Figure S5**).

## DISCUSSION

Three-year follow-up data from the IMPACT registry indicate positive outcomes following SAVR using the INSPIRIS RESILIA bioprosthesis in a real-world prospective cohort. This study builds on the growing body of evidence regarding the use of bioprostheses with RESILIA tissue following SAVR, particularly the COMMENCE trial, which demonstrated excellent mid- and long-term durability in a selective patient population.^11^^,^^12^^,^^14^ Other recent studies have echoed these findings in various clinical contexts,^8–10^^,^^19^ with some conducted in broader, real-world patient populations.^20^

In the IMPACT registry, the 3-year overall survival rate was 91.0%, the valve-related survival rate was 97.6%, and the in-hospital mortality rate was 1.4%. These outcomes align with (1) the ACTIVIST trial 3-year survival rate of 95.2% and in-hospital mortality rate of 1.5% (mean age 74 years)^19^; (2) real-world evidence from Europe showing a 5-year survival of 93.2% with a cardiovascular survival rate of 97.2% (mean age 60 years)^20^; and (3) a German cohort of patients demonstrating a 3-year survival of 87.7% with no valve-related mortality.^10^ The observed 3-year survival reflects all-comers real-world population with a broad range of medical histories and comorbidities and complex valve pathology, including a substantial proportion of bicuspid aortic valves and concomitant procedures.

Valve-related dysfunction was low at 3 years and was assessed using VARC-3 echocardiographic criteria,^18^ which may be considered more sensitive than previous SVD definitions that required clinical end-points such as reintervention and morphological evidence.^21^ In the present study, moderate HVD was observed in 6 patients (FFE 97.7%) and severe HVD in 1 patient (FFE 99.5%), which aligns with 3-year moderate (FFE 99.7) and severe (FFE 100%) HVD rates of a large real-world European registry.^20^ In the COMMENCE trial (mean age 67.0 years), SVD (stage ≥2) was 1.8% at 5 years (VARC-3),^12^ which agrees with the valve durability seen in the present IMPACT cohort.

Although the IMPACT population was younger (63.4 years) than the cohort enrolled in the COMMENCE trial, a greater proportion had advanced symptoms at baseline. Despite this, both studies demonstrated a significant improvement in the NYHA class following SAVR, which is also consistent with the sustained haemodynamic improvement from baseline to 3 years shown in other studies reporting favourable functional and haemodynamic performance of the INSPIRIS RESILIA valve.^13^^,^^15^ Notably, in this study, outcomes were similar in patients <60 and ≥60 years, suggesting that factors beyond age may likely affect post-operative survival and valve durability.

The IMPACT registry demonstrates that the INSPIRIS RESILIA valve performs well in a diverse population. The growing demand for durable tissue valves reflects the need to balance patient preferences, risk, and long-term outcomes, particularly as younger patients increasingly seek alternatives to lifelong anticoagulation that are compatible with active lifestyles. The forthcoming 5-year follow-up will provide further insights into the mid- to long-term outcomes.

### Limitations

Although this study presents positive 3-year outcomes of patients who underwent SAVR with the INSPIRIS RESILIA AV as part of the IMPACT registry, certain limitations need to be acknowledged. For instance, it is descriptive and without a comparator group, preventing direct comparisons with other valves, and 3 years is insufficient to infer long-term outcomes. Further, data on alternative tissue valve use during the enrollment period were not prospectively collected, and roughly 20% of enrolled patients were lost to incomplete follow-up data or administrative discontinuation (ie, not lost to study-related adverse outcomes), both of which limit contextual interpretation. Nevertheless, present data provide meaningful insights and real-world patient outcomes, as patients with multiple pre-existing comorbidities were included in the analysis.

## CONCLUSION

The 3-year follow-up data from the IMPACT registry add to real-world evidence on the INSPIRIS RESILIA valve, providing data on survival, haemodynamic performance, and early safety.

## Supplementary Material

ivag056_Supplementary_Data

## Data Availability

All relevant data within this manuscript will be shared upon reasonable request to the corresponding author.
