# Supplementary material for: Three-Year Outcomes After Surgical Aortic Valve Replacement With a Bioprosthetic Valve from the Multi-Centre IMPACT Registry
Source: Interdiscip Cardiovasc Thorac Surg. 2026 Mar 5;41(3):ivag056. doi: 10.1093/icvts/ivag056 (PMC12981659; doi:10.1093/icvts/ivag056)
Supplement: ivag056_Supplementary_Data [file ivag056_supplementary_data.zip › 25-Feb-2026_100344_IMPACT_Supplement.docx]

# Supplementary figures/tables

**Supplementary Figure 1:** Distribution of valve sizes

| 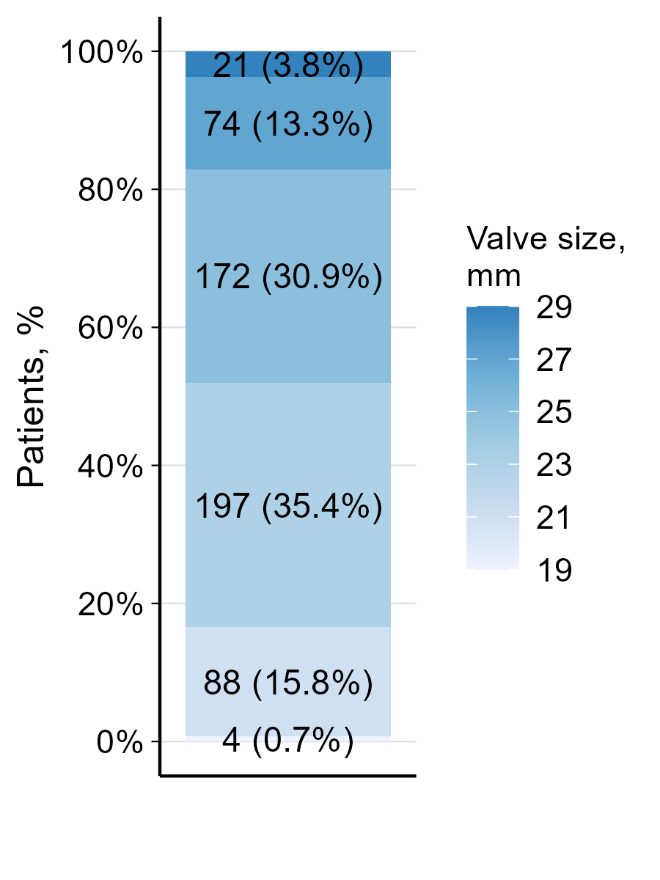 |
| --- |

**Supplementary Figure 2:** Three-year paravalvular leak grade changes

| 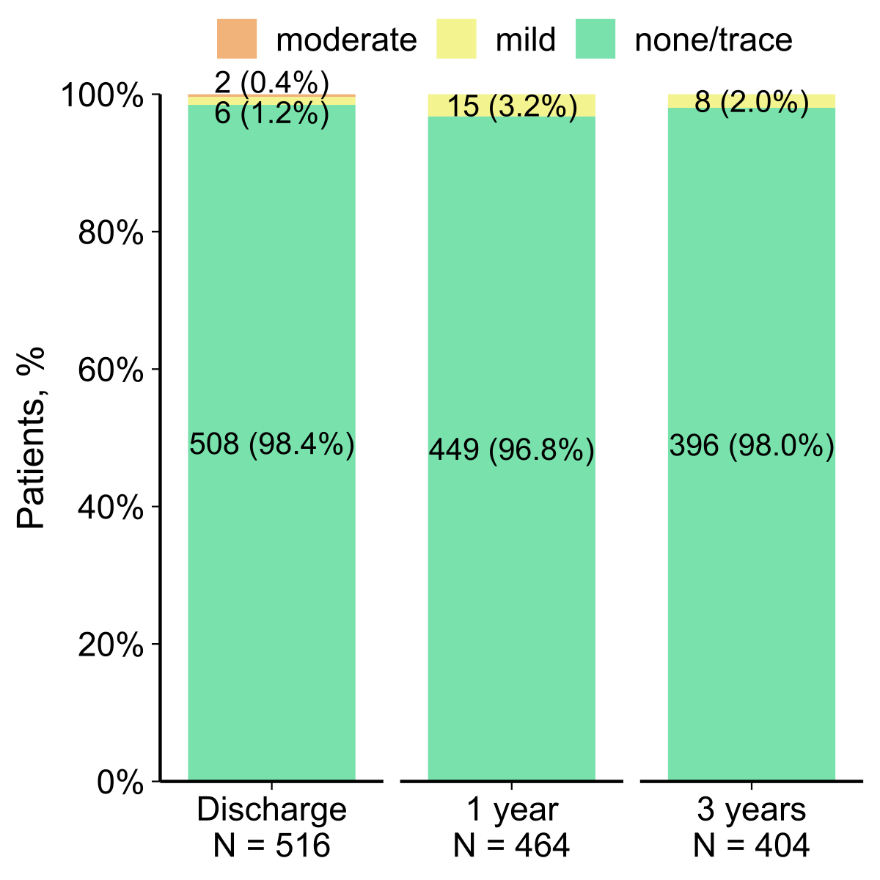 |
| --- |

**Supplementary Figure 3:** Three-year mean transvalvular pressure gradient change

| 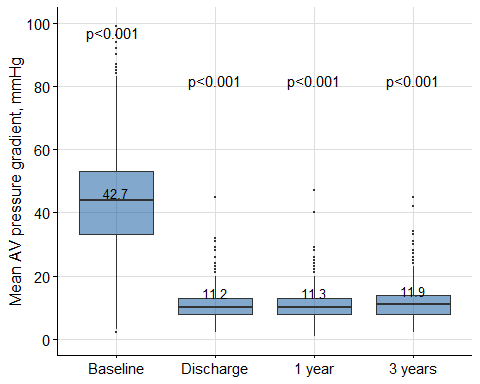 |
| --- |

Legend: AV, aortic valve.

**Supplementary Figure 4:** Three-year left ventricular mass index changes

| 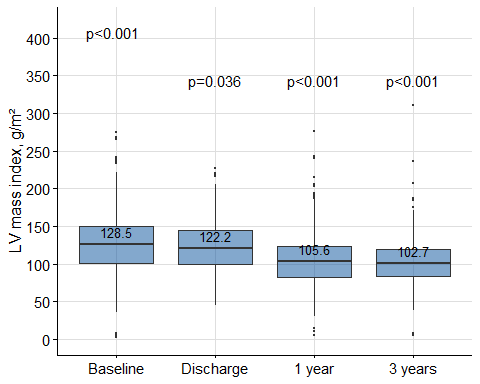 |
| --- |

Legend: LV, left ventricular.

**Supplementary Figure 5:** Three-year repeat procedure cumulative incidence by age group

| 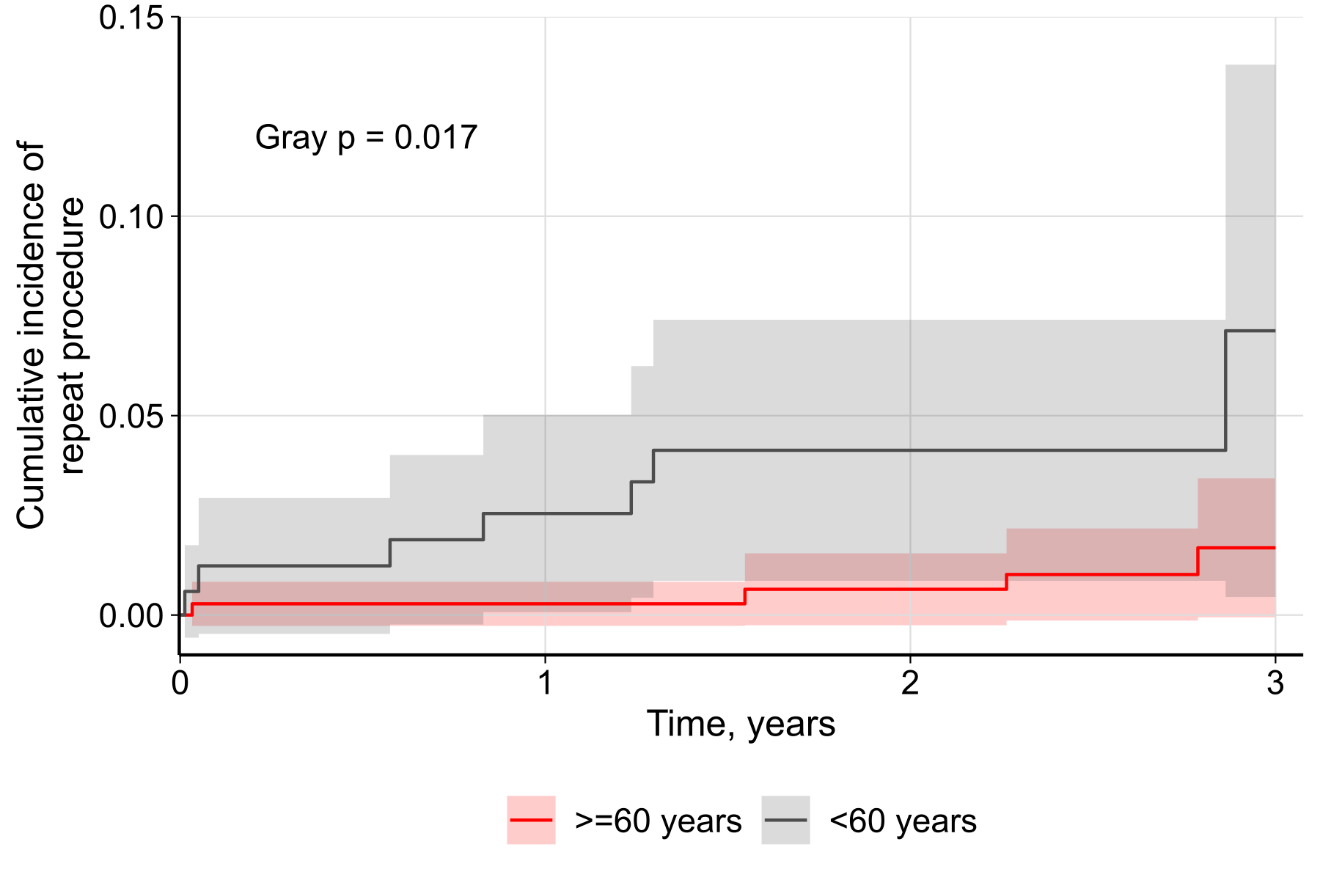 |
| --- |

**Supplementary Table 1:** Three-year clinical outcomes divided by age

|  | **Patients under 60, n = 170** | | | | **Patients above or equal 60, n = 386** | | | |  |
| --- | --- | --- | --- | --- | --- | --- | --- | --- | --- |
|  | **Total number of events, n** | **Early events (≤30 days), n (% of 170)** | **Late events (up to 3 years), n (linearised rate/valve years)** | **Freedom from events at 3 years, % (95% CI)** | **Total number of events, N** | **Early events (≤30 days), n (% of 386)** | **Late events (up to 3 years), n (linearised rate/valve years)** | **Freedom from events at 3 years, % (95% CI)** | **log-rank p-value** |
| All-cause mortality | 10 | 2 (1.2) | 8 (2.2) | 92.4 (88.0, 97.1) | 23 | 6 (1.6) | 17 (2.1) | 90.6 (85.7, 95.8) | 1.000 |
| Cardiovascular-related mortality | 5 | 2 (1.2) | 3 (0.8) | 96.7 (93.8, 99.6) | 16 | 6 (1.6) | 10 (1.2) | 93.6 (89.2, 98.2) | 0.516 |
| Valve-related mortality | 2 | 0 (0.0) | 2 (0.6) | 98.5 (96.5, 100.0) | 10 | 4 (1.0) | 6 (0.7) | 97.1 (95.4, 98.9) | 0.296 |
| Endocarditis | 6 | 1 (0.6) | 5 (1.4) | 93.5 (87.2, 100.0) | 8 | 0 (0.0) | 8 (1.0) | 97.5 (95.9, 99.2) | 0.309 |
| Valve thrombosis | 0 | 0 (0.0) | 0 (0.0) | 100.0 (100.0, 100.0) | 0 | 0 (0.0) | 0 (0.0) | 100.0 (100.0, 100.0) | 1.000 |
| Stroke/transient ischemic attack | 8 | 6 (3.5) | 2 (0.6) | 95.1 (91.8, 98.5) | 16 | 13 (3.4) | 3 (0.4) | 95.6 (93.5, 97.8) | 0.782 |
| Stroke | 5 | 4 (2.4) | 1 (0.3) | 96.9 (94.3, 99.6) | 12 | 12 (3.1) | 0 (0.0) | 96.8 (95.1, 98.6) | 0.896 |
| Valve-related dysfunction | 3 | 2 (1.2) | 1 (0.3) | 98.1 (96.0, 100.0) | 4 | 0 (0.0) | 4 (0.5) | 98.3 (96.5, 100.0) | 0.485 |
| Requirement for repeat procedure | 7 | 2 (1.2) | 5 (1.4) | 92.9 (86.5, 99.7) | 4 | 1 (0.3) | 3 (0.4) | 98.3 (96.6, 100.0) | **0.016** |
| Permanent pacemaker implant | 11 | 9 (5.3) | 2 (0.6) | 93.6 (89.9, 97.5) | 23 | 15 (3.9) | 8 (1.0) | 93.4 (90.8, 96.1) | 0.809 |
| HVD stage 2 | 3 | 0 (0.0) | 3 (0.8) | 97.1 (94.0, 100.0) | 3 | 0 (0.0) | 3 (0.4) | 98.0 (95.6, 100.0) | 0.302 |
| HVD stage 3 | 0 | 0 (0.0) | 0 (0.0) | 100.0 (100.0, 100.0) | 1 | 0 (0.0) | 1 (0.1) | 99.3 (98.0, 100.0) | 0.501 |

Legend: Age <60: the sum of valve years is 400.8; age ≥60: the sum of valve years is 913.3; HVD, haemodynamic valve deterioration.
